# Unsupervised machine learning with Cluster Analysis in patients with rheumatoid arthritis: insights in CV risk phenotypes from a multicentre Italian cohort

**DOI:** 10.1007/s11739-026-04478-9

**Published:** 2026-09-02

**Authors:** Vincenzo Venerito, Elena Bartoloni, Gian Luca Erre, Matteo Piga, Garifallia Sakellariou, Ombretta Viapiana, Andreina Manfredi, Elisa Gremese, Fabiola Atzeni, Francesca Romana Spinelli, Fabio Cacciapaglia

**Affiliations:** 1 Rheumatology Unit, Department of Precision and Regenerative Medicine and Ionian Area (DePReMeI), Università Degli Studi “Aldo Moro”, Bari, Italy; 2 Rheumatology Unit, Department of Medicine and Surgery, University of Perugia, Perugia, Italy; 3 Dipartimento Di Scienze Mediche, Chirurgiche E Sperimentali, Università Degli Studi di Sassari, Sassari, Italy; 4 Rheumatology Unit, AOU Cagliari, and Department of Medical Sciences and Public Health, University of Cagliari, Cagliari, Italy; 5 Department of Internal Medicine and Therapeutics, University of Pavia, Pavia, Italy; 6 Istituti Clinici Scientifici Maugeri IRCCS Pavia, Pavia, Italy; 7 Rheumatology Unit, Department of Medicine, University and Azienda Ospedaliera Universitaria Integrata of Verona, Verona, Italy; 8 Rheumatology Unit, Azienda Ospedaliera Universitaria Policlinico of Modena, Modena, Italy; 9 Rheumatology and Clinical Immunology, IRCCS Humanitas Research Hospital, Rozzano, Italy; 10 Department of Biomedical Sciences, Humanitas University, Pieve Emanuele, Italy; 11 Rheumatology Unit, Department of Experimental and Internal Medicine, University of Messina, Messina, Italy; 12 Rheumatology Unit, Department of Medical and Cardiovascular Sciences, Sapienza University of Rome, Rome, Italy; 13 Rheumatology Service, ‘‘F. Miulli’’ Hospital, Department of Medicine and Surgery Libera, Università del Mediterraneo (LUM) ‘‘G. de Gennaro’’, Casamassima, Bari Italy

**Keywords:** Rheumatoid arthritis, Cardiovascular risk, Major cardiovascular events, Machine learning, Unsupervised clustering, Risk stratification, SCORE2

## Abstract

Rheumatoid arthritis (RA) patients face increased cardiovascular (CV) risk, yet existing 10-year estimation tools often underperform. This study evaluated whether unsupervised machine learning (uML) clustering can identify distinct RA patient phenotypes with varying major cardiovascular events (MACE) potentially improving risk stratification. A cross-sectional, multi-center cohort of RA patients meeting the 2010 ACR/EULAR classification criteria and without prior CV events was enrolled in January 2019 and followed-up for MACE incidence over 5 years. Clinical and demographic data, including traditional CV risk factors and SCORE2 estimates, were collected. Factor Analysis of Mixed Data (FAMD), a generalization of principal component analysis for mixed data types, was applied in Python (ver.3.9) using Prince (ver.0.7.1). Missing data observations were excluded. Bootstrapped eigenvalue distribution determined the number of FAMD components for clustering, followed by Hierarchical Clustering on Principal Components using Ward’s criterion and Euclidean distance. An XGBoost model assessed feature importance, while ANOVA and Chi-square tests evaluated cluster differences. Among 951 RA patients (mean age 61.8 ± 10.5 years; 81.1% female; median disease duration 120 months), 9 MACE occurred (incidence: 1.89/1000 patient-years). Four clusters emerged: Cluster 1 (high HAQ, steroid use, high cholesterol), Cluster 2 (low disease activity, BMI, diabetes, hypertension, SCORE2), Cluster 3 (predominantly male, < 10-year disease duration, csDMARDs; no MACE cases), and Cluster 4 (longer disease duration, hypertension, high triglycerides, lipid-lowering agents, TNFi; highest MACE incidence).uML clustering identified distinct RA phenotypes, complementing SCORE2 for improved CV risk stratification and aiding in recognizing patients at elevated MACE risk.

## Introduction

Rheumatoid arthritis (RA) patients have an increased risk of all-cause mortality compared to the general population, with cardiovascular disease (CVD) being the leading cause of death in these patients, accounting for about 40% of their mortality [1]. Traditional cardiovascular (CV) risk factors do not fully explain the higher incidence of CVD in RA patients, and chronic systemic inflammation is likely the link between RA and the increased CV risk [2].

Most CV events could be prevented by early detection and management of modifiable CV risk factors and ensuring adequate preventive strategies. Estimating CV risk is crucial, as it helps in assessing the individual’s likelihood of experiencing a CV event within a defined timeframe, thereby informing treatment decisions [3].

Many international authorities have proposed different tools to assess CV risk in the general population, supporting healthcare professionals in designing tailored intervention strategies [4]. In this setting, the European Society of Cardiology guidelines have recommended the Systematic Coronary Risk Evaluation (SCORE) algorithm since 2016. This algorithm, updated in 2021, draws on data from 12 European cohort studies and comprises distinct charts designed for implementation in high-, moderate-, and low-risk countries [5]. However, prediction algorithms perform better in populations similar to those from which they were derived, but their accuracy may decrease when used in groups with different characteristics. Furthermore, it is important to note that none of the predictive CV risk algorithms encompasses all relevant risk factors but are usually limited to a selected few; they often do not account for the duration of exposure to different risk factors, thus showing overall low sensitivity with a lack of consensus regarding the optimal risk-scoring tool to use in clinical practice [6].

In recent years, significant progress has been made in data mining technology, an essential and valid tool for constructing risk assessment models. In this setting, artificial intelligence (AI), incorporating machine learning (ML), represents a branch of computer science dedicated to creating computational systems capable of executing tasks traditionally associated with human intelligence [7]. AI is a promising approach for CVD risk prediction, demonstrating superior data-processing capability, fewer constraints, and enhanced performance in analyzing big data sources. Recent applications of AI in CV research have been promising and have provided interesting preliminary results in risk stratification, diagnosis, treatment, and prognosis of CVD in non-RA populations [8, 9]. On the other hand, very few studies have explored the role and possible application of AI-based systems in CV risk estimation, prognosis, and risk stratification in patients with RA [10, 11]. Integrating genomic and radiomic-based biomarkers within AI models holds significant potential for accurately detecting the risk and severity of CVD in these patients, pending further research and validation [11]. In particular, ML- and deep learning-based algorithms have demonstrated good feasibility in characterizing carotid plaque tissue and composition by ultrasonography or other high-resolution imaging techniques, thus allowing a more reliable stratification of CV risk in RA patients [12, 13]. However, to our knowledge, AI-based analysis of RA clusters and phenotypes according to the risk of developing major adverse cardiovascular events (MACEs) has not yet been undertaken.

Thus, this study aimed to apply unsupervised machine learning (uML) to discover inherent and distinct RA phenotypes with varying frequencies of MACEs.

## Patients and methods

Since January 2019, a multicentre cohort of RA patients fulfilling the 2010 ACR/EULAR classification criteria [14], without previous CV events, enrolled at the 10 Italian University Centers of the Cardiovascular Obesity and Rheumatic Diseases (CORDIS) study group, on behalf of the Italian Society for Rheumatology (SIR), has been longitudinally followed until December 2023 for the occurence  of MACEs.

Comprehensive clinical and serologic data were collected at enrollment according to standardized definitions. Clinical and demographic features, including traditional CV risk factors, the 10-year CV risk according to the SCORE2 algorithm and MACEs, were recorded at study entry. Specifically, traditional CV risk factors included smoking status (current, former, never), body mass index (BMI), systolic and diastolic blood pressure values, fasting lipid profile of total cholesterol, high-density lipoprotein cholesterol (HDL-C), low-density lipoprotein cholesterol (LDL-C), and triglycerides. Hypertension was defined as a medical history of hypertension or current use of blood pressure-lowering drugs according to guidelines for hypertension management. Hyperlipidemia was defined as the use of lipid-lowering medications and/or total cholesterol ≥ 200 mg/dl (5.7 mmol/L). Diabetes mellitus was diagnosed upon confirmation of patient history and/or the documented use of oral or injectable glucose-lowering medications or insulin.

At entry, the SCORE model was applied using the calculator available on the website [15]. Then, in 2021, the CV risk of each enrolled patient was ‘recalibrated’ according to the updated version of the SCORE2 model to estimate 10-year fatal and non-fatal CVD risk in Italian individuals [5].

The occurrence of MACE was defined as acute coronary syndrome (ST- and non-ST elevation myocardial infarction, coronary revascularization, unstable angina), ischemic stroke or peripheral artery disease (with and without revascularization procedures). All CV events were retrieved by reviewing medical charts; patients with previous MACE were excluded from the analysis.

RA-specific features such as disease duration, Disease Activity Score 28 (DAS28) by C-reactive protein (CRP), and Clinical Disease Activity Index (CDAI), as measures of disease activity, and Health Assessment Questionnaire (HAQ) disability index as a functional index were collected [16–18]. The serologic status of rheumatoid factor (RF) and anti-citrullinated peptide antibodies (ACPA) was obtained using local assays.

Finally, information on ongoing antihypertensive and lipid-lowering therapies, disease-modifying anti-rheumatic drugs (DMARDs), including conventional synthetic (cs), biologic (b), and/or targeted synthetic (ts) DMARDs, and glucocorticoids (mean weekly dose since diagnosis and the current daily dose of prednisone or equivalent), was collected.

During the follow-up period, comprehensive clinical data were collected at least once per year through detailed clinical histories during scheduled patient visits. Any MACE occurring during the follow-up was accurately tracked.

The study followed the Good Clinical Practice guidelines and the latest Declaration of Helsinki. The local Ethical Committees approved the study as part of the GISEA Registry Protocol (ClinicalTrials.gov ID NCT01543594; approval number DG-624/2012). Written informed consent was collected from the patients involved.

### Data analysis

The research methodology employed a comprehensive uML approach to uncover natural patterns within patient data without predefined labels or outcomes. Unlike supervised approaches that require predetermined categories, uML clustering analysis allows the data to reveal intrinsic patient subgroups based on complex interactions among multiple clinical variables. This approach is particularly valuable in heterogeneous conditions like RA, where traditional classification methods might miss subtle but clinically significant patient phenotypes.

The analysis began with careful pre-processing of clinical and laboratory variables; this ensured data quality by excluding cases with critical missing information and standardizing features to address unit differences through Z-score normalization.

At the core of the analysis, Factor Analysis of Mixed Data (FAMD) served as a sophisticated dimensionality reduction technique. FAMD was particularly valuable because it could simultaneously process quantitative and qualitative variables, effectively generalizing principal component analysis for mixed datasets. This analysis was implemented using Python 3.9 with the Prince 0.7.1 library, with the number of components determined through bootstrapped eigenvalue distribution analysis [19].

The reduced dataset underwent hierarchical clustering analysis using Ward’s criterion and Euclidean distance metrics. The elbow method guided the selection of an optimal number of clusters, carefully balancing granularity against the risk of overfitting. Cluster separation was visualized using the first two principal components, demonstrating clear between-cluster separation while maintaining reasonable within-cluster variability, even in the presence of potential outliers.

To interpret and validate the clustering results, the analysis employed extreme gradient boosting (XGBoost) as a post hoc tool. The XGBoost classifier was configured with standard hyperparameters: learning rate (eta) of 0.1, maximum tree depth of 6, minimum child weight of 1, subsample ratio of 0.8, and colsample_bytree of 0.8. The number of estimators was set to 100, with early stopping rounds of 10 to prevent overfitting. L1 (alpha) and L2 (lambda) regularization terms were set to 1, and the objective function was set to ‘multi:softmax’ for multiclass classification. XGBoost was trained using the identified clusters as dependent variables against all other attributes, generating feature importance rankings based on F1 scores. This step provided crucial insights into which variables most strongly influenced cluster formation and separation.

The statistical robustness of the clustering solution was verified through a combination of ANOVA for continuous variables and chi-square tests for categorical variables, ensuring that the identified groups represented meaningfully distinct patient categories. This comprehensive approach allowed for the discovery of more profound levels of disease categorization while maintaining methodological rigor and clinical relevance.

## Results

A total of 951 RA patients without prior CV events and complete dataset were included in the analysis. At entry, the mean age was 61.8 ± 10.5 years, 81.1% were female (*n* = 771), and the median RA disease duration was 120 months (95% CI 102–126). Table 1 summarizes demographic, clinical, and laboratory findings of the whole cohort of patients.

**Table 1:** Patient characteristics by cardiovascular risk phenotype derived from unsupervised clustering

	Overall	Hierarchical cluster labels				*P* value
		Cluster 1	Cluster 2	Cluster 3	Cluster 4	
Patients, *n*. (%)	951	293 (30.8)	195 (20.5)	279 (29.3)	184 (19.3)	< 0.0001
Age, years, mean SD	61 ± 10.5	66.1 ± 9.5	54.7 9.8	58.8 ± 9.3	67.2 ± 8.4	< 0.0001
Gender, male, *n*. (%)	180 (18.9)	12 (4.1)	21 (10.8)	107 (38.4)	40 (21.7)	< 0.0001
Previous CV event	0					
Rheumatoid Factor positive - n. (%)	627 (65.9)	200 (68.3)	124 (63.6)	175 (62.7)	128 (69.6)	0.315
ACPA, positive, *n*. (%)	604 (63.5)	185 (63.1)	137 (70.3)	164 (58.8)	118 (64.1)	0.087
Smoker, *n*. (%)	118 (19)	52 (17.7)	37 (19)	57 (20.4)	35 (19)	0.881
BMI, Kg/m2, mean (95%CI)	25.6 (21.3–28.3)	25.6 (25–26.2)	23.1 (22.5–23.9)	24.6 (24.2–25.2)	26.4 (25.7–27.3)	< 0.0001
Disease duration, months, median (95%CI)	120 (102–126)	144 (120–156)	96 (84–108)	99 (96–118)	156 (144–180)	< 0.0001
csDMARDs, *n*. (%)	643 (67.6)	186 (63.5)	123 (63.1)	206 (73.8)	128 (69.6)	< 0.0001
Glucocorticoids, *n*. (%)	410 (43.1)	180 (61.4)	72 (36.9)	84 (30.1)	74 (40.2)	< 0.0001
HAQ, median (95%CI)	0.75 (0.25–0.75)	1 (1–1.25)	0.375 (0.25–0.6)	0.4 (0.25–0.5)	0.875 (0.625–1)	< 0.0001
Hypertension, *n*. (%)	384 (40.4)	179 (61.1)	10 (5.1)	38 (13.6)	157 (85.3)	< 0.0001
Antiplatelet, *n*. (%)	60 (6.3)	25 (8.5)	7 (3.6)	8 (2.9)	20 (10.9)	0.0007
Anticoagulant, *n*. (%)	5 (0.5)	2 (0.7)	0	2 (0.7)	1 (0.5)	0.714
Dyslipidemia, *n*. (%)	592 (62.3)	232 (79.2)	110 (56.4)	121 (43.4)	129 (70.1)	< 0.0001
Lipid-lowering drugs , *n*. (%)	182 (19.1)	82 (28)	16 (8.2)	27 (9.7)	57 (31)	< 0.0001
CRP mg/l, median (95%CI)	3.2 (3–3.7)	4.2 (2.1–5)	2.3 (2–3)	3 (2.7–3.8)	4 (3–4.5)	0.0002
CDAI, median (95%CI)	6 (6–7)	7 (6–8)	4.5 (3–5.5)	6 (5–7)	6.1 (6–8)	0.01
DAS-28 ESR, median (95%CI)	3.1 (3.02–3.23)	3.25 (3.1–3.5)	2.72 (2.42–2.86)	3.06 (2.87–3.22)	3.42 (3.19–3.66)	< 0.0001
Disease duration > 10 years, *n*. (%)	433 (45.5)	161 (54.9)	63 (32.3)	86 (30.8)	123 (66.8)	< 0.0001
Diabetes, *n*. (%)	56 (5.9)	23 (7.8)	6 (3.1)	9 (3.2)	18 (9.8)	0.003
Anti-hypertensive drugs t, *n*. (%)	370 (38.9)	172 (58.7)	10 (5.1)	36 (12.9)	152 (82.6)	< 0.0001
Systolic Blood Pressure, median (95%CI)	125 (122–130)	130 (130–130)	120 (112–120)	120 (120–125)	135 (130–140)	< 0.0001
Diastolic Blood Pressure, median (95%CI)	77 (75–80)	80 (75–80)	70 (70–75)	75 (75–79)	80 (80–80)	< 0.0001
Total Cholesterol (mg/l), median (95%CI)	207 (203–210)	218 (213–225)	207 (199–213)	195 (188–198)	208 (199–215)	< 0.0001
HDL Cholesterol (mg/dl), median (95%CI)	60 (59–61)	63 (61–66)	66 (64–68)	55 (53–57)	55 (53–58)	< 0.0001
Tot Cholesterol/HDL chol., median (95%CI)	3.35 (3.29–3.42)	3.35 (3.23–3.45)	3.04 (2.96–3.16)	3.45 (3.33–3.59)	3.57 (3.42–3.73)	< 0.0001
Triglycerides (mg/dl), median (95%CI)	95 (92–99)	105 (99–112)	79 (71–85)	87 (82.93)	106 (100–113)	< 0.0001
KDOQI Stage III-IV-V, n. (%)	4 (0.4)	1 (0.3)	1 (0.5)	1 (0.4)	1 (0.5)	0.176
Headache, *n*. (%)	7 (0.7)	2 (0.7)	1 (0.5)	3 (1.1)	1 (0.5)	0.677
Psychiatric disorder (severe), *n*. (%)	7 (0.7)	5 (1.7)	1 (0.5)	1 (0.4)	0	0.121
Anti-psychotic treatment, *n*. (%)	8 (0.8)	6 (2)	1 (0.5)	1 (0.4)	0	0.052
Erectile dysfunction, *n*. (%)	0	0	0	0	0	
New CV event, *n*. (%)	9 (0.9)	2 (0.7)	0	0	7 (3.8)	0.0001
SCORE2, median (95%CI)	5.2 (4.8–5.6)	6.2 (5.6–6.7)	3.1 (2.7–3.6)	4.6 (4–5)	8.2 (7–9.3)	< 0.0001
TNFi, *n*. (%)	237 (24.9)	0	0	123 (44.1)	114 (62)	< 0.0001
JAKi, *n*. (%)	42 (4.4)	37 (12.6)	0	0	5 (2.7)	< 0.0001

*CV* cardiovascular, *RF* rheumatoid factor; *ACPA* anti-citrullinated peptides antibodies, *NMI* body mass index, *csDMARDs* conventional synthetic Disease-Modifying Anti-Rheumatic Drugs, *HAQ* Health Assessment Questionnaire, *CRP* C-reactive protein, *CDAI* Clinical Disease Activity Indexm, *DAS28* Disease Activity Score 28, *HDL* high-density lipoprotein, *KDOQI* Kidney Disease Outcomes Quality Initiative, *SCORE2* Systematic Coronary Risk Evaluation 2, *TNFi* tumor necrosis factor inhibitors, *JAKi* Janus Kinases inhibitors

**Fig. 1:**
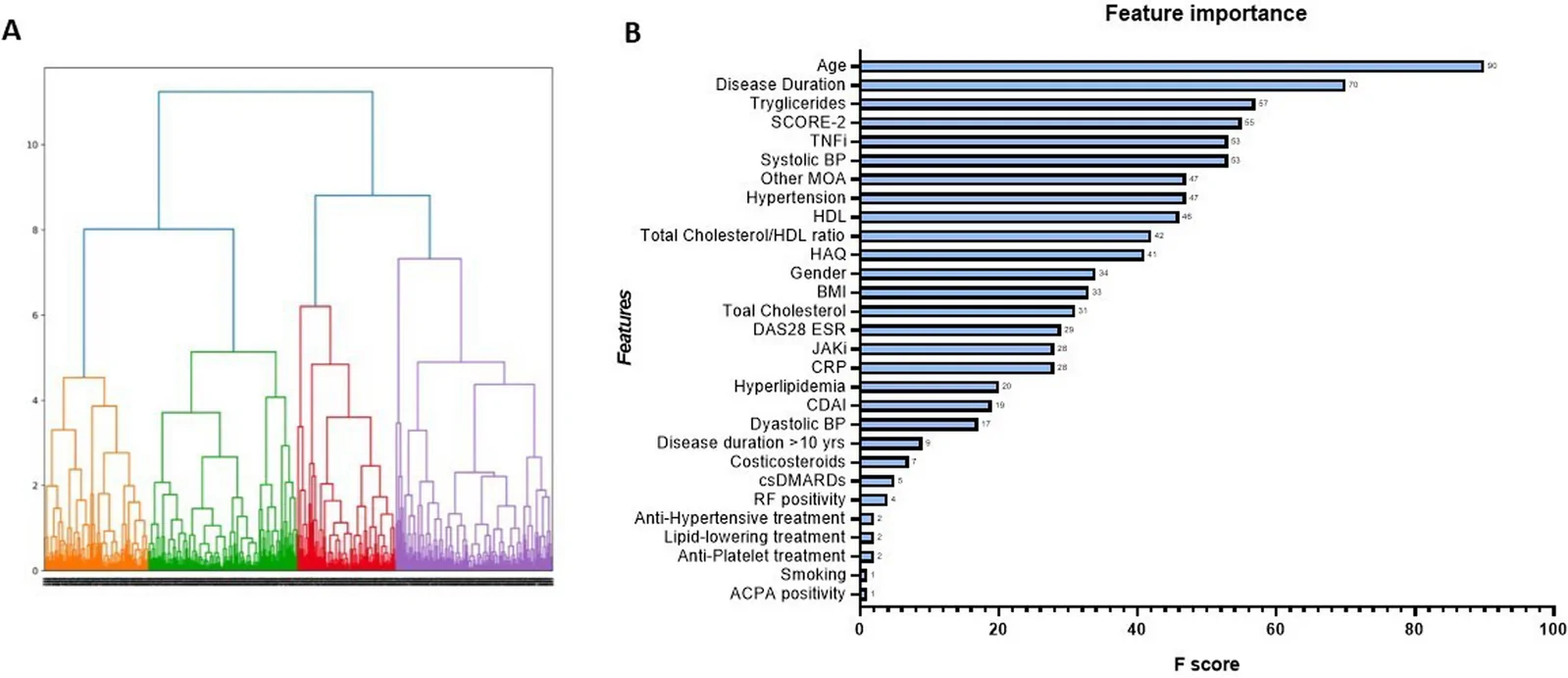
Agglomerative hierarchical clustering analysis with definition of the dendrogram (**A**). Feature importance analysis and identification of the relevant factors for clustering (**B**). *TNFi* tumor necrosis factor inhibitors, *BP* blood pressure, *MOA* mode of action, *HDL* high-density lipoprotein, *HAQ* Health Assessment Questionnaire, *BMI* body mass index, *DAS28* Disease Activity Score 28, *JAKi* Janus Kinases inhibitors, *ESR* erythrocyte sedimentation rate, *CRP* C-reactive protein, *CDAI* Clinical Disease Activity Index, *csDMARDs* conventional synthetic Disease-Modifying Anti-Rheumatic Drugs, *RF* rheumatoid factor, *ACPA* anti-citrullinated peptides antibodies

An agglomerative hierarchical clustering analysis was performed, with the number of clusters defined by the dendrogram (Fig. 1A). A four-cluster solution was selected to avoid excessive fragmentation while balancing discriminatory capacity and interpretability. Feature importance analysis (Fig. 1B) identified age, disease duration, triglycerides serum level, and SCORE2 as the most influential variables for clustering, all showing statistically significant differences across groups.

Key features achieving statistical significance used to drive the cluster differentiation are reported in Table 1. The four clusters comprise 293, 195, 279, and 184 patients, respectively; demographic, clinical, and RA-related characteristics varied significantly among them.

Cluster 1 included the highest proportion of females and was marked by higher disease activity according to CDAI, greater functional disability, and higher systemic inflammation according to C-reactive protein values and, consequently, a higher rate of glucocorticoid use. Moreover, patients of Cluster 1 were more frequently prescribed JAK inhibitors.

Cluster 2 included the youngest patients, more often treated with non-TNF inhibitor bDMARDs. Cluster 3 consisted mostly of males, who were often treated with csDMARDs, and had shorter disease duration (< 10 years). Finally, Cluster 4 included a subset of patients characterized by older age, longer disease duration, a higher proportion of subjects with disease duration > 10 years, seropositivity for RF, higher disease activity according to DAS28, and the highest rate of anti-TNF prescriptions.

In terms of traditional CV risk factors, Cluster 4 showed the highest prevalence of hypertension and diabetes, the lowest lipid levels—and the largest proportion of patients taking lipid-lowering medications—and the highest BMI. Cluster 1 exhibited the highest lipid levels and Cluster 2 the lowest prevalence of hypertension and diabetes.

Due to the highest prevalence of traditional CV risk factors and the older age, median 10-year CV risk scores (SCORE2) were highest in Cluster 4 (8.2%, 95% CI 7.0–9.3%), followed by Cluster 1 (6.2%, 95% CI 5.6–6.7%) and Cluster 3 (4.6%, 95% CI 4.0–5.0%). Cluster 2 had the lowest estimated risk (3.1%, 95% CI 2.7–3.6%).

**Fig. 2:**
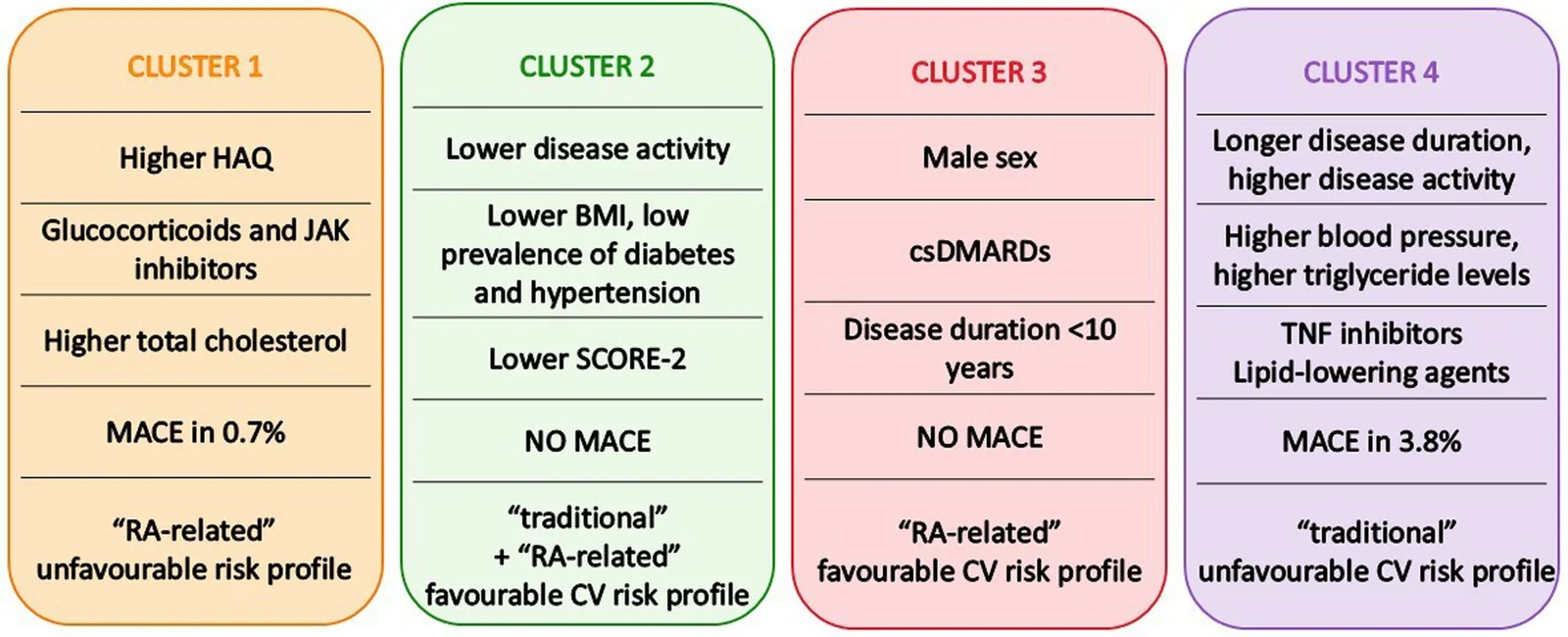
Traditional and RA-related CV risk factors in the clustering analysis. *HAQ* Health Assessment Questionnaire, *MACE* major cardiovascular events, *RA* rheumatoid arthritis, *BMI* body mass index, *CV* cardiovascular, *TNF* tumor necrosis factor

Summarizing the hierarchical characteristics of the four clusters (Fig. 2), Cluster 1 included patients with the highest levels of disability and disease activity, the greatest proportion of glucocorticoid users, and the highest total cholesterol levels. Cluster 2 was characterized by the lowest disease activity, BMI, prevalence of diabetes and hypertension, and the lowest SCORE2 values. Cluster 3 had a higher proportion of male patients with disease duration < 10 years, and predominant use of csDMARD therapy. Cluster 4 comprised older patients with longer disease duration, elevated disease activity, higher systolic blood pressure and serum triglyceride levels, and the largest proportion of individuals treated with lipid-lowering medications and anti-TNF drugs.

The incidence rate (IR) of new MACE in the overall cohort was 1.89 per 1000 person-years, with notable differences across clusters. Cluster 4 had the highest IR of 7.61 per 1000 person-years, reflecting the convergence of ‘traditional’ and ‘RA-related’ CV risk factors, whichaccounted for the worst CV risk profile. Cluster 1 had an IR of MACE of 1.37 per 1000 person-years mainly driven by ‘RA-related’ CV risk factors. No new MACE occurred in patients of Clusters 2 and 3, which showed cardiovascular risk profiles characterized by either a combination of low traditional and RA-related risk factors, or predominantly low RA-related risk factors alone, respectively.

## Discussion

The results of this study support the utility of uML-derived clustering, combined with the SCORE2 algorithm, for identifying distinct clinical characteristics of RA patients with different CV risk profiles. Within the paradigm of precision medicine, reliable identification of patient subsets at a higher risk of CVD is highly desirable to personalize preventive strategies and target specific therapeutic approaches [20]. The uML approach offers several advantages in this context. First, uML allows for unbiased pattern recognition without predetermined outcomes, potentially uncovering previously unrecognized patient subgroups; second, it can handle complex, multidimensional data and identify non-linear relationships that traditional statistical methods might miss. This approach enables a more nuanced assessment of CV risk, particularly in patients with RA, by identifying specific disease phenotypes relevant to CV health and improving the predictive accuracy of existing CV risk models in recognizing patients at highest risk of MACE.

Notably, our study is the first to demonstrate the utility of uML clustering analysis for identifying distinct disease phenotypes and varying CV risk profiles within a large Italian multicentre RA cohort. The hierarchical clustering revealed significant differences among patient groups, reinforcing the importance of personalized treatment approaches in managing the disease [20]. Notably, hierarchical clustering identified Cluster 4, characterized by a significantly higher occurrence of MACE during follow-up. This cluster exhibited the most unfavorable combination of traditional and disease-related risk factors, including older age, longer disease duration, higher disease activity, elevated systolic blood pressure, and increased serum triglyceride levels. Moreover, Cluster 4 included the highest proportion of patients treated with lipid-lowering agents and anti-TNF, probably as a consequence of higher disease activity. Interestingly, the study allowed the identification of a patient group, included in Cluster 1, characterized by higher systemic inflammation, disease activity and functional disability, and a consequent higher need for glucocorticoid therapy. Intriguingly, these factors, likely reflecting the heightened inflammatory burden in this group, may partly explain the observed highest prevalence of dyslipidemia and elevated total cholesterol levels. Indeed, RA-related inflammatory mechanisms accounting for the “lipid paradox” may be associated with an altered lipid profile, and RA treatments can affect cholesterol metabolism, thereby suggesting the importance of regular monitoring of the lipid profile during the different disease activity stages to initiate  lipid-lowering therapy according to validated recommendations [21–23].

The significant age differences observed across the clusters likely reflect varying stages of disease progression and the potential accumulation of comorbidities in older patients. The older mean age of patients included in Cluster 4 suggests a group with long-lasting disease and, possibly, a greater prevalence of comorbidities, thus needing more intensive management strategies. In contrast, the younger age of patients included in Cluster 2 might reflect a group with earlier diagnosis and less severe RA manifestations, as demonstrated by lower DAS28 scores. Given the known influence of gender on RA phenotype, treatment response, and the distribution of traditional CV risk factors (leading to differing 10-year CVD risk), our findings further underscore the importance of considering sex-specific risk factor profiles in CV risk screening for RA patients [24]. In this study, the gender disparity, particularly the lower percentage of males in Cluster 1 (4.1%) and the higher percentage in Cluster 3 (38.4%), may influence disease characteristics, treatment response, and the specific CV risk profile. Moreover, Cluster 3 included a higher proportion of smokers (20.4%), suggesting the urgent need for targeted interventions to reduce smoking-related risks and improve overall health outcomes in this subgroup.

The variation in glucocorticoid and biological drug prescriptions across clusters highlights intriguing differences in disease management approaches. For instance, the high prevalence of glucocorticoid use in Cluster 4 might reflect attempts to control more severe and stabilized manifestations of the disease; on the other hand, chronic glucocorticoid use represents a risk factor for CV events in these patients [25–27]. In contrast, we observed higher use of JAK inhibitors in Cluster 1. Interestingly, this cluster included the lowest proportion of males and smokers and was characterized by higher disease activity, inflammatory burden, and, consequently, glucocorticoid use. Notably, despite the higher disease activity, the use of JAK inhibitors in this cluster did not correspond  to a higher occurence of MACE, contrary to what the results of the ORAL Surveillance study might have suggested [28–30].

The significant differences in MACE occurrence across clusters underscore the importance of individualized CV risk assessment and management in RA patients [20]. In this setting, the higher risk of MACE observed in Cluster 4 surely suggests the need for more accurate CV monitoring and intervention in patients with the worst risk profile due to a combination of traditional and disease-related factorsincluding older patients, with longer disease duration and active disease, and a higher prevalence of specific traditional CV risk factors, such as hypertension and diabetes. The high prevalence of hypertension and diabetes in Cluster 4 further compounds the CV risk, highlighting the need for comprehensive management of these comorbidities [31]. Also, the CV risk assessment score, which was highest in Cluster 4, underlines the need for tailored risk mitigation strategies based on individual patient risk profiles.

On the other hand, other clusters might benefit from different preventive strategies.

Finally, the results of this study also suggest that the frequency of MACE in RA is not strictly connected to the traditional CV risk factors. In these patients, the risk seems to be more closely linked to other disease-related features. Indeed, longer disease duration and higher disease activity emerged as the most critical factors contributing to the increased CV risk in RA patients, in association with hypertension and dyslipidemia.

In conclusion, unsupervised ML clustering analysis is a powerful technique for discovering patterns in data, but it comes with some limitations and challenges. In uML, there are no predefined labels or outcomes against which to compare the clustering results, making it difficult to assess the quality and accuracy of the clusters without external validation. The present cluster analysis suggests future research directions for the early identification of patients with high CV risk and for supporting the timely implementation of CV preventive measures. This study provides the first hierarchical clustering of a large multicenter RA cohort according to different CV risk profiles in combination with specific disease features. In a recent survey, unsupervised clustering analysis by Self-Organizing Maps was utilized to analyze circulating molecular profiles in pre-selected cohorts with different CV risk profiles [32].

Interestingly, the derived risk prediction models may incorporate cluster-specific data into their items, enhancing their accuracy and utility in clinical practice. Developing clinical decision support systems based on clustering analysis can aid healthcare providers in making data-driven treatment decisions, ultimately improving patient care quality. The implementation of uML in clinical practice also presents both opportunities and challenges. While it offers powerful insights for patient stratification and personalized medicine, it requires substantial computational resources and expertise to implement effectively. Furthermore, integrating uML-derived insights into clinical decision-making necessitates careful validation and standardization across different healthcare settings. However, some limitations should be acknowledged. uML results can be sensitive to feature selection and scaling, and the interpretation of clusters requires clinical expertise to ensure meaningful conclusions. Additionally, the stability of clusters over time needs further validation, and longitudinal studies to track changes in patient clusters are required to provide deeper insights into the progression of RA and the long-term effectiveness of personalized treatment strategies. Moreover, we cannot exclude that other clustering methods may give different results. Therefore, future research should integrate additional multidimensional data, such as genetic, environmental, and lifestyle factors, to refine patient phenotyping and improve the precision of personalized medicine approaches.

Despite these limitations, the potential benefits of uML in identifying distinct patient phenotypes and predicting cardiovascular risk make it a valuable tool for advancing personalized medicine in RA care. By elucidating the diverse phenotypes and associated CV risks, our findings pave the way for more comprehensive and effective management strategies tailored to individual patient profiles. The goal is to enhance RA patients’ quality of life and clinical outcomes through personalized, data-driven healthcare.

## Data Availability

The data used in this study are not publicly available due to ethical restrictions, including patients’ confidentiality agreements and institutional data protection policies. De-identified data supporting the findings of this study are available from the corresponding author upon reasonable request.
